# Controlling Malaria on the Road to Elimination: A View from 10 Years Later

**DOI:** 10.4269/ajtmh.24-0629

**Published:** 2024-10-29

**Authors:** Richard W. Steketee

**Affiliations:** Independent consultant, Bethesda, Maryland

Ten years ago (November, 2014), Kent (Carlos C.) Campbell authored a brief opinion piece for a planned submission to the American Journal of Tropical Medicine and Hygiene.^1^ He was retiring soon, and this would have been his last formal comment in the field of malaria. Alas, he did not submit it at that time, and it gathered dust on a computer hard disk thereafter. Kent died this year at age 80.^2^^,^^3^ His thoughts in 2014 continue to have relevance for today and our future.

Kent had an illustrious career in public health and working against malaria. Beginning in 1972, he worked for more than 20 years at the U.S. Centers for Disease Control and Prevention (CDC), leading the Malaria Branch for more than a dozen years. He also gained the perspectives of academia (starting the University of Arizona College of Public Health), UN agencies (consultancies with WHO and helping UNICEF for two years to build its malaria program), foundations (as an early malaria consultant in the Bill and Melinda Gates Foundation [BMGF]), non-governmental organizations (more than a decade with PATH leading the Malaria Control and Elimination Partnership in Africa [MACEPA]), and endemic countries (living in El Salvador in 1973–1976 and many collaborations with African nations over the years). Throughout his career, he was active in the American Society of Tropical Medicine and Hygiene (ASTMH) and served as councilor and then as President in 2007; in 2012, he received the Joseph Augustin Le Prince Medal recognizing his outstanding work in the field of malariology. He trained, mentored, and learned from many people along the way.

What led Kent to put his comments on paper? Kent began his malaria career approximately three years after the end of the WHO-led Global Malaria Eradication Program, when two interventions (vector control with indoor residual spraying [IRS] and wide-scale treatment [with chloroquine and other drugs] to clear parasites) met their match with mosquito and parasite resistance.^4^^,^^5^ The next nearly three decades saw a move to “malaria control” as a global strategy^6^ that focused on malaria “burden-reduction” – reducing illness, severe disease, and death. This interval included a resurgence of science and field work to better describe malaria epidemiology in Africa^7^^,^^8^ and seek to demonstrate the efficacy and effectiveness of new tools – new drugs, diagnostics, and insecticides, and methods for delivery with insecticide treated mosquito nets (ITNs).^9^^,^^10^^,^^11^ Given malaria’s outsized contribution to global child disease and death and the availability of new tools, enthusiasm gradually returned for national malaria program support. Starting in late 1998, the multilateral Roll Back Malaria Partnership renewed engagement with malaria-endemic nations. Investment followed, and by 2005, The Global Fund, U.S. President’s Malaria Initiative, World Bank Booster Program, BMGF, and others including malaria-endemic countries were supporting national malaria programs. Investments were increasing annually and, by 2007, BMGF leadership put “elimination” and “eradication” (i.e., taking local infection transmission-reduction to zero) at the forefront of the discussion as a goal for the more than 100 malaria-endemic countries.^12^ Over the next several years, many experts highlighted both opportunities and challenges; however, ending malaria transmission remained the goal.^13^

As a pediatrician, Kent championed the challenging struggle to eliminate malaria, especially in Africa and for African children. He focused on balancing good science and information with support for programs to deliver a package of effective interventions to achieve population-wide impact through reduction of transmission and disease burden. Following the 2007 BMGF announcement encouraging a focus on elimination, Kent published on goals for malaria elimination.^14^^,^^15^ But, in 2014, he worried that we might forget our history and back away from critically important work and the focused goal of malaria elimination.

Global funding for malaria had increased from US$960 million in 2005 to US$2.5 billion by 2014, but it stayed essentially unchanged from 2009 onward.^16^^†^ Coupled with steady population and cost of living increases, the investment per-population-at-risk had been decreasing each year. It would be easy to back away from elimination as a goal when progress was stalling – we had done this before.

But, as Kent points out in his opinion piece, the pathway to elimination includes good case management that reduces severe disease and death, but also absolutely requires transmission-reduction (i.e., turning off the tap of new infections). This pathway is a continuum, in which early gains can be seen in the reduction of morbidity and mortality, especially in the highest risk populations of young African children, and long-term gains are seen as elimination.

What has transpired in the last decade since Kent’s 2014 draft? An immediate change arrived in a publication by Bhatt and colleagues^17^ showing best modeled estimates, whereby approximately two-thirds of the improvement in malaria burden between 2000–2015 was attributable to vector control interventions (primarily ITNs and IRS) that specifically reduced malaria transmission – demonstrating the critical requirement of prevention of new infections as part of the continuum toward burden reduction and elimination.

The elimination agenda has progressed substantially in the last decade. Since 2000, 25 countries achieved three consecutive years of zero cases of indigenous malaria.^18^ While four countries sought and received WHO malaria elimination certification between 2000–2014, 12 countries have certified as malaria-free since 2014 (including El Salvador in 2021—with Kent as a co-author nearly 50 years after he worked there).^19^ Among the 86 countries that were malaria-endemic in 2000, 46 (including those achieving zero cases) recorded an annual incidence <1 case per 1,000 population between 2000–2019, a threshold associated with a subsequent 10-year horizon to achieve elimination.^20^^,^^21^ Unfortunately, 34 countries (31 in sub-Saharan Africa) have greater than 1 million cases annually, and their citizens suffer approximately 95% of the global malaria illness and death burden.^18^ In these countries malaria transmission typically remains saturated, with far too many infectious mosquito bites per person each day.

Funding continues at a stable level but is decreasing per-population-at-risk. Some tools have been improved. For example, ITNs and IRS evidence and guidance now includes new insecticides or enhancements that partially mitigate the threat of insecticide resistance.^22^^,^^23^ Improvements are evolving, with progress toward new diagnostics,^24^ new drugs,^25^^,^^26^ and new information on safety and targeting of drugs in specific populations.^27^ Two new vaccines have been approved for use in young children at high risk – albeit for reduction of burden, but not transmission.^28^^,^^29^ Advances in other vaccines that have transmission reduction potential is encouraging.^30^ Early findings with malaria-specific monoclonal antibodies suggest that they may offer both disease and transmission reduction.^31^^,^^32^ Studies of vector control measures to complement existing ITNs and IRS are underway.^33^^,^^34^^,^^35^ However, investment in science for new tools, especially those focused on transmission reduction, has also stagnated or fallen since 2017,^18^ and this represents an enormous risk for the future.

Kent worried about the young children in Africa. In our resource-constrained world, targeting the highest risk populations typically takes precedence. The 2018 emphasis on “high-burden-high-impact” countries and the subsequent “sub-national tailoring” of intervention strategies has extended this targeting process.^36^^,^^37^ In the Yaoundé Conference of 2024, African Ministers of Health were asked to commit to “accelerated reduction of malaria mortality”^38^^,^^39^ – a laudable effort, but at risk of emphasizing specific burden-reduction approaches. Africa was left out of the initial WHO Global Malaria Eradication Program. It would be a tragedy to repeat history and move our current goal away from elimination, and instead pretend that control in the face of continued malaria transmission could be a clear and measurable goal. Some will say that it is best to be respectful of our limitations, financial and otherwise, and that we should welcome a first step of commitment to control, first reducing child deaths, and focusing on elimination later. But as Kent offered, “later” could be far too late for a great number of young children in Africa and their siblings, mothers, and fathers who deserve better.

## Footnotes

^†^
Annual WHO World Malaria Reports began highlighting this plateau in 2017, and it is now standard language that investment and the fight against malaria is stalled.
